# Pharmacological Evaluation of Selected Medicinal Plants Used in the Management of Oral and Skin Infections in Ebem-Ohafia District, Abia State, Nigeria

**DOI:** 10.1155/2018/4757458

**Published:** 2018-07-04

**Authors:** Blessing O. Oyedemi, Sunday O. Oyedemi, Johnson V. Chibuzor, Ifeoma I. Ijeh, Roger M. Coopoosamy, Ayobami O. Aiyegoro

**Affiliations:** ^1^Department of Plant Science and Biotechnology, Michael Okpara University of Agriculture, Umudike, Nigeria; ^2^Department of Biochemistry, Michael Okpara University of Agriculture, Umudike, Nigeria; ^3^Department of Nature Conservation and Ethnobotany, Mangosuthu University of Technology, P.O. Box 12363, Jacobs, Durban 4026, South Africa; ^4^GI Microbiology and Biotechnology Unit, Agricultural Research Council, Animal Production Institute, Irene, Pretoria 0062, South Africa

## Abstract

Oral and skin infections contribute significantly to the global health challenges responsible for the current trend of increased morbidity and premature death. The purpose of this study was to document medicinal plants used in the management of oral and skin infections in Ebem-Ohafia Local Government Area (LGA), Abia State, and to characterize the* in vitro* antioxidant and antibacterial activity. The thin layer chromatography (TLC) profiling of ten of the selected folklore medicine was carried out using a various solvent system of different polarity index. The antioxidant capacity of the plant extracts was evaluated using chemical-based methods, and its antibacterial effect was investigated using disc diffusion and microdilution methods. Sixty-one plant species belonging to 26 families were discovered, and the most frequently cited species are Euphorbiaceae (18.03%), Fabaceae (11.47%), and Asteraceae (11.47%). All the plant extracts showed a promising free radical scavenging activity and efficient ferric reducing antioxidant power in a concentration-dependent manner possibly due to their richness in polyphenol with TLC profiling showing maximum three bands of phytochemicals. Also, the plant extracts exhibited a mild to weak antibacterial activity against our panel of bacterial strains having MIC values ranging from 256 to > 512 *μ*g/ mL reflected in their zone of inhibition at 10 *μ*g/disc. The data obtained for* Breynia nivosa* (BN),* Eleusine indica* (EI),* Cassia alata* (CA),* Chromolaena odorata* (CO), and* Acalypha hispida* (AH) extracts substantiate the traditional use of these herbal remedies in the region and open the possibility for the development of cheaper and affordable drugs in the treatment of oral and skin infections. Further studies are needed to identify active ingredient with strong antibacterial and antioxidant capacities along with their molecular mechanisms.

## 1. Introduction

Oral health is a state of being free from the mouth and facial pain, throat cancer, oral infection, and sores that limit human capacity in biting, chewing, smiling, and speaking as well as psychosocial well-being [1]. Owing to the environmental pollution, the concentration of heavy metals such as Ni, Cr, Zn, Hg, and Pb in most food materials and water is rapidly increasing which severely affects both oral and general health condition. For example, dental caries, periodontitis, and throat cancer have been recognised as the most prevalent noncommunicable diseases associated with heavy metals, globally affecting school-aged children and a huge majority of adults [2]. In Nigeria, the rate of deep periodontal pocket is between 15 and 58% (aged 15 years) while the prevalence of unmanaged dental caries is high raising alarm for odontogenic infection. Falsetta and colleagues [3] highlighted that poor oral hygiene and frequent consumption of sugars create bacterial adhesion to tooth surfaces via mineralization and biofilm formations caused by glucosyl and fructosyl transferases. The same authors indicated an adverse effect of higher concentration of fluoride in drinking water in some developing countries that is responsible for the cause of fluorosis and dental caries regardless of age and sex. The oral cavity exposed to carcinogens that may advance into precancerous lesions and malignant disorders like leukoplakia, submucous fibrosis, and dental caries resulting from the imbalance in the levels of free radicals generation and natural antioxidants in the saliva [4]. There is a growing consensus that administration of natural antioxidants to the oral cavity reduces free radicals, oxidative stress, and oral inflammation triggered by nicotine, alcohol, and other toxins introduced into the mouth [5].

The skin, being the largest organ in the body, guards the underlying muscles, bones, ligaments, and internal organs interfaces with the environment, as well as protecting the body against pathogenic microbes [6]. Notwithstanding, infectious skin diseases such as skin abrasions, burns, acne, dermatitis, and sebaceous cysts have become everyday incidences and constitute a reoccurring health problem that affects all human age groups [6]. Over the years, bacterial skin disorders such as impetigo, ecthyma, folliculitis, eczemas, and urticarias are one of the most common causes of hospital visits in the southeastern part of Nigeria. Overcrowding, malnutrition and humidity, heat, food, and medication allergies cause some of these skin diseases, especially among the school children in urban and rural settings [2]. Microbial invasion of the skin surface forms a biofilm that confers a drug tolerance on the bacteria and capacity to escape host immune response [7]. The discovery of antibiotic resistance genes in several microorganisms, quorum sensing, and horizontal gene transfer has limited treatment of skin and soft tissue infections and thus could pose a serious health challenge in a region where there is limited access to microbiological laboratory facilities and antimicrobial agents [8]. The global need for effective alternative medicines for oral and skin infections with minimal side effects and economical use is increasing due to the rapid growth of antibiotic resistance and opportunistic infections in immunocompromised patients [9].

Plant-derived products are now gaining recognition worldwide currently recommended for primary healthcare, but only a few plants have received rigorous scientific investigation. Medicinal plants are natural products used since time immemorial for the treatment of various human diseases. All over the world, the citizen and health practitioners are now starting to rely on herbal medicines as a substitute for scientifically proved therapies [10]. Herbal products contained secondary metabolites that are synthesized through the pentose phosphate, shikimic acid, and phenylpropanoid pathways. These compounds play a significant defensive role against pathogenic bacteria and oxidative stress caused by abiotic stress in plant [11]. Currently one of the strategies used to treat skin and oral infections is the use of natural products from plant origin perhaps due to the perception that long-term use of western medicine induces severe complications. Consequently, many Nigerian patients, in particular, those living in the rural areas, have resorted to seeking relief via traditional healers and herbalists who administer herbal drugs in the management of human diseases. Considering the rate at which the vegetation gets depleted and the current focus on safe complementary and alternative medicine to synthetic drugs, there is, therefore, the need to document the indigenous knowledge of these plants and to search for more plants with therapeutic potential in the treatment of bacterial infections and oxidative stress.

There is a paucity of literature in traditional medicine used in the management of orofacial and skin infections in Nigeria. Our recent ethnobotanical survey conducted in Ebem-Ohafia LGAs in the southeastern part of Nigeria revealed sixty-one plant species commonly used in the management of oral and skin infections. Despite the acclaimed traditional use of these botanicals as therapeutic agents, there is little scientific evidence to support their folkloric use. Therefore, this study aimed at investigating the antioxidant capacity of these herbal remedies in terms of free radical scavenging, reducing power, and total phenolic and flavonoid content in consonance with the TLC profiling. The antibacterial effect on some clinically isolated bacteria associated with oral and skin infections was also investigated.

## 2. Materials and Methods

### 2.1. Study Area

Ebem-Ohafia is a town in Ohafia LGA of Abia state in the southeastern part of Nigeria situated between 5° 38′N and 7° 50′ East and encompasses over twenty-six hometowns with a population of over 300, 000 from the last population census in 2006. Ebem community is a part of the tropical rainforest characterized by dry and rainy season with a total annual rainfall of over 1400 mm and an annual temperature range of 23°C to 32°C. The people of the region practice subsistence farming under local government agencies policy. The inhabitants of Ebem use herbal drugs either alone or in combination with other medicines in the treatment of human diseases. Most of the people in Ebem are rural dwellers; hence they use plant-based therapies in the management of ailments that include oral and skin infections.

### 2.2. Ethnobotanical Survey

The ethnobotanical survey was conducted between December and May 2016 using a well-structured questionnaire administered to the participants with indigenous knowledge of plants utilized in the area. The set questions contained the local diagnosis of oral and skin diseases, the name of plant samples used for the treatment, methods of preparation, and duration of treatment, side effects, and mode of administration. The people interviewed consisted of women and men both married and unmarried at the age of 30 to 65 with little education qualification.

### 2.3. Plant Collection and Identification

The leaves of* Ipomoea involucrata* (II),* Acalypha hispida* (AH),* Breynia nivosa* (BN),* Jatropha curcas* (JC),* Chromolaena odorata* (CO),* Macrolobium macrophyllum* (MM),* Baphia nitida* (BNI),* Burkea africana* (BA),* Cassia alata* (CA), and the root of* Eleusine indica* (EI) were collected after the interview. The plants were identified by their local names and authenticated by Mr. Oriaku Williams and Professor Gabriel Osuagwu from the Department of Plant Science and Biotechnology, College of Natural Sciences, Michael Okpara University of Agriculture, Umudike in Abia state, Nigeria. The vouchers specimens (VICJON 1–10) were prepared and deposited at the departmental herbarium. The preparations and mode of intake of the herbal remedies are described in Table 1.

### 2.4. Sample Preparation

We collected leaves and roots of the ten plants that were frequently mentioned in the survey and oven-dried at 40°C for 72 h while the root of EI was dried longer for seven days. The dried plant materials were pulverized to a fine powder using an electric blender and stored in an airtight container for further use. Thirty grams (30 g) of dried powdered materials were extracted with 150 mL of 100% methanol for 48 h on a mechanical shaker (Stuart Scientific Orbital 20.2, SOSI, Essex, UK) and the extracts were filtered using Buchner funnel and Whatman Number 1 filter paper. The filtrate was concentrated using a rotary, evaporated at 40°C to recover the solvent, and air-dried in a fume chamber to give a yield ranging from 4.5 to 8.3 g.

### 2.5. Thin Layer Chromatography (TLC) Profile

Thin layer chromatography is a simple method for analyzing a complex mixture of compounds based on the distance travelled. The plant extracts (1 mg/mL) dissolved in methanol and spotted on the plate coated with silica gel 60 F254 as a stationary phase. The slurry was prepared by dissolving 15 g of silica gel 60 F254 in 30 mL of distilled water and immediately poured into the plate. The plates were air-dried overnight. About 10 *μ*L of the plant extracts was gently loaded on the base of the plate (5 cm above) using the capillary tube. The plates were allowed to develop in chromatographic tanks consisting of three different solvents (mobile phase) chloroform: methanol: acetic acid (5:4:1) until they reach 3/4th of the TLC plate. The TLC plate was removed and allowed to dry, the spots were detected by iodine vapour, and retention factor (R_f_) was calculated using the equation: R_f_ = distance travelled by the components/distance travelled by the solvent.

### 2.6. Total Phenolic Content (TPC) Assay

The total phenolic content present in these extracts was quantified by the Folin Ciocalteu reagent (FCR), using the method of Ghaffari et al. [12]. Briefly, 0.2 mL of the plant extract (2 mg/mL) was added to the reaction mixture consisting of 1 mL of 10% v/v FCR and 0.8 mL of Na_2_CO_3_ (0.075 mg/mL) to give a final concentration of 0.2 mg/mL of each extract. The resulting mixture was incubated at 45°C with shaking for 15 min and the absorbance measured at 765 nm. A standard curve was prepared by mixing methanol solution of gallic acid (0.2 mL; 0.025 - 0.400 mg/mL) with 1 mL of 10% v/v FCR and sodium carbonate (0.8 mL, 0.075 mg/mL). The experiment was carried out in triplicate, and the results presented as mean values with standard deviation (± SD). The TPC value was expressed as milligrams of gallic acid equivalent (GAE) per g of dried sample. It was calculated using the formula: T = C×V/M, where T is the TPC (mg/g) of extract, in GAE; C is the concentration of gallic acid from the calibration curve; V is the volume of the extract, mL; M is the dry weight (g) of the leaf or root powder from which the extract was obtained.

### 2.7. Total Flavonoid Content (TFC) Assay

The concentration of flavonoids in the plant extracts was determined via aluminum colorimetric assay method [13]. Briefly, 1 mL of 2% w/v AlCl_3_ was prepared in 100%v/v methanol added to 1 mL of the sample solution. A yellow colour formation after incubation at room temperature for 1 h was measured at 420 nm using an AJI-C03 UV_VIS spectrophotometer. The standard curve for TFC was obtained using quercetin as a standard drug under the same procedure described in TPC determination. The TFC was calculated using the formula: T = C×V/M, where T is the TFC (mg/g) of extract, in QE; C is the concentration of quercetin established from the calibration curve; V is the volume of the extract, mL; M is the dry weight (g) of the leaf powder from which the extract was obtained. The TFC was present in the extracts calculated as mg/g of quercetin equivalent (QE).

### 2.8. Ferric Reducing Antioxidant Power (FRAP) Assay

The ability of plant extracts to reduce ferric (Fe^3+^) to ferrous (Fe^2+^) form was evaluated following the method described by Yen and Chen [14] with slight modification. A volume of 0.3 mL of different concentrations (0.025 - 2 mg/mL) from plant extract, BHT, ascorbic acid, and rutin prepared in distilled water was mixed with reacting mixture consisting of 2.5 mL of 0.2 M phosphate buffer (pH 6.6) and 2.5 mL of K_3_Fe(CN)_6_ (1% w/v). The resulting mixture was incubated at 50°C for 20 min followed by addition of 2.5 mL of TCA (10% w/v). After vigorous shaking, 2.5 mL of the solution was mixed with 2.5 mL of distilled water and 0.5 mL of FeCl_3_ (0.1% w/v) left incubated at room temperature for 5 min and then the absorbance was measured at 700 nm against a blank sample (without extract).

### 2.9. Free Radical Scavenging Activity Assays

The free radical scavenging potential of plant extracts was measured in vitro by the 1, 1′-diphenyl-1-picrylhydrazyl (DPPH) described by Tariq et al. [15]. The assay was experimented by reacting 1.6 mL of 0.135 mM DPPH dissolved in 100%v/v methanol with 0.4 mL of various concentrations (0.078 - 1 mg/mL) of methanol crude extracts. The reaction mixture was vortexed thoroughly and left in the dark at room temperature for 30 min. The absorbance of the mixture was measured at 517 nm after 2 min. The method of Re et al. [16] was adopted to determine ABTS radical scavenging activity of the plant extracts. The ABTS radical solution was generated by mixing two stock solutions of 7 mM ABTS and 2.4 mM potassium persulphate in the same ratio and allowing the solution to react for 12 h at room temperature in the dark. The resulting solution was diluted with methanol to obtain an absorbance of 0.706 units at 734 nm. A volume of 1 mL of various concentrations (0.03125 – 1 mg/mL) of the plant extracts react with 2.5 mL of ABTS radical solution in the dark for 15 min and was later measured the absorbance. The percentage inhibition of DPPH or ABTS radical scavenging activity by the plant extracts was calculated as {(Abscontrol – Abssample)}/(Abscontrol) × 100, where Abscontrol is the absorbance of DPPH or ABTS + methanol; Abssample is the absorbance of DPPH or ABTS radical + sample extract/standard. Here, the concentration of the extracts needed to decrease the absorbance of DPPH or ABTS radical by 50% was calculated. Rutin (Sigma-Aldrich, ≥ 94%, HPLC grade) at the same working concentrations of the plant extracts was used as reference drug.

### 2.10. Bacterial Strains

All bacterial strains were cultured on nutrient agar slopes and were incubated for 24 h at 37°C before the MIC. An inoculum turbidity equivalent to a 0.5 McFarland standard (1×10^7^ CFU/mL) was prepared in normal saline for each test organism and then diluted 1:100 in Mueller-Hinton broth just before injection of the plates. Standard strains used in the study included* S. aureus* and* E. coli*, which were a gift from Dr. EO Ekundayo (Department of Microbiology, Michael Okpara University of Agriculture), and clinically isolated strains of* K. pneumoniae* and* P. mirabilis* were gotten from Department of Microbiology, Federal Medical Centre (FMC) Umuahia, Abia State.

### 2.11. Antibacterial Susceptibility Using Disc Diffusion Method

The microbial growth inhibition potential of the plant extracts was determined by using the agar disc diffusion method [17]. The plant extracts were dissolved in 10% v/v DMSO (Merck UK Ltd) to a final concentration of 2 mg/mL. The nutrient agar plate was streaked with the standardized test bacterial inoculum over the surface evenly, and 2 mg/disc of the plant extracts transferred onto sterile filter disc papers (about 6 mm) containing the test compounds was placed on the agar surface in the Petri dishes. 100 *μ*g/disc of standard antibiotic ciprofloxacin was used as control and 100 *μ*L DMSO/disc served as negative control. The plates were then incubated at 37°C for 24 h. Generally, the antimicrobial extracts diffuse into the agar and inhibit the growth of the test bacteria. All tests were done in duplicate and diameter zones of inhibition of growth were measured using a meter rule from the edge of each dish after the incubation period.

### 2.12. Minimum Inhibitory Concentration (MIC) Assays

We followed the method of Smith et al. [18] to determine the MIC of methanol crude extracts of selected botanicals against clinical isolates of bacteria associated with skin and oral infection. A volume of 100 mL of sterile Mueller-Hinton broth (Oxoid, Basingstoke, UK) containing 20 mg/L of Ca^2+^ and 10 mg/L of Mg^2+^ was dispensed into a 96-well microtitre plate (Nunc; 0.3 mL total volume per well). All antibacterial agents were dissolved in dimethyl sulphoxide (DMSO) and diluted in Mueller-Hinton broth to give a stock solution. Then, 100 *μ*L of the antibacterial agent stock solution (2000 mg/L) was serially diluted into each well and 100 mL of the bacterial inoculum added to each well to give a final concentration range of 0.024 - 1 mg/L. All procedures were performed in duplicate and the plates were incubated for 18 h at 37°C. Briefly, 20 mL of a 5 mg/L methanol solution of 3-[4,5-dimethylthiazol-2-yl]-2,5-diphenyltetrazolium bromide (MTT) (Sigma-Aldrich Ltd, South Africa) was added to each well and then incubated for 30 min. Blue colouration indicated bacterial growth. The MIC was recorded as the lowest concentration at which no colour change was observed.

### 2.13. Statistical Analysis

Data analysis was done in Microsoft Excel to obtain descriptive statistics. Means values were separated by the Duncan multiple tests using SAS. The different levels of significance within the groups were analyzed using one-way analysis of variance (ANOVA). Values were considered significant at* P* <0.05.

## 3. Results and Discussion

### 3.1. Ethnobotanical Survey

Modern drugs are expensive and supply to a remote area may be irregular, hence resulting in overreliance of traditional methods of healing as a primary source of healthcare [19]. The rural communities of Ebem-Ohafia region of southeastern Nigeria depend on herbal remedies to meet their domestic and health needs. During our ethnobotanical survey, a total of 30 respondents including the elders, herbalists, and traditional healers were interviewed in the study area. Majority of the people (22) were aged 30–65 years, dominated by males (25), and one-third (10) had a primary school education while 15 had secondary education and others informal education. A total of 61 medicinal plant species belonging to 26 families were identified in the treatment of oral and skin diseases as shown in Table 1. Twenty-seven of them are used in the management of oral infections and twenty-six plants for the management of skin infection while eight plants are used in the management of both infections.  Table 2 shows the list of family, number of the plant species used, and their percentage occurrence. The most represented plant family is the Euphorbiaceae having 18.03% of the plant species followed by Asteraceae and Fabaceae with 11.47% each [20]. Euphorbiaceae is a large family of flowering plants with 300 genera and about 7500 species found useful in the curing of some common human diseases. Asteraceae is a large family of flowering plants having 32, 913 species and 1911 genera and has been found to possess diverse biological effects. Fabaceae is a family of cosmopolitan distribution, with approximately 730 genera and 19,400 species richness at a global level [20]. The leaves (60%) recorded the most commonly used plant part followed by the root (16%) and then stem bark (12%). None of the respondents indicated any possible side effects caused by any of these plants listed in Table 1. The most common methods of preparation include infusion, decoction, and maceration. These herbalists or traditional healers often collect the plants from the field and dry and crush them into powder form before storing in bottles to prevent patients from identifying the plants used for their treatment. Most healers refused to provide information without payment whereas others thought to provide information on the plant use is disrespect of their traditional belief. Furthermore, the majority of the plants mentioned lack scientific data to substantiate their acclaimed folkloric use. From this work, it is evident that medicinal plants play a significant role in the primary healthcare of the people, and conservation of the indigenous knowledge of this folklore medicine is crucial for the innovations of future drug discovery and possible herbal plants trade in the region [19]. On the contrary, we observed imprecise dosage, low hygiene standards, hiding of healing methods, and lack of patients' record as a significant setback to traditional health practitioners in the region.

### 3.2. Thin Layer Chromatography (TLC) Profiling

Thin layer chromatography remains the most common useful technique by researchers as a quick and simple method to resolve the constituents of a crude extract followed by advanced extraction methods to purify the active ingredients. Previous studies reported that specific secondary metabolites in plant exhibit a wide range of antioxidant and antibacterial properties [21]. TLC was formed on a glass sheet coated with silica gel. After the sample is applied to the plate, a solvent system (mobile phase) is drawn up via capillary action on the TLC plate at different mobility rate depending on the chemical compositions. The separated spots with different colours were visualized by the UV light projection onto the glass sheet. The variation of coloured spots detected in the crude extracts indicates the presence of different phytochemicals that may be responsible for the pharmacological effects of selected plants use in the management of oral and skin infections as acclaimed by the traditional healers. We quantified the results by measuring the distance travelled by the plant compounds divided by the total distance travelled by the mobile phase. From Table 3, it is apparent that mobile phase 1 consisting of chloroform and ethanol in the volume ratio of 6:4 had a better separation than mobile phase II containing toluene, ethyl acetate, and acetic acid in the volume ratio of 5:4:1. The methanol extract of AH in both mobile phases had the highest number of three bands with R_f_ values of 0.667, 0.833, and 0.989 for mobile phase I and R_f_ values of 0.381, 0.648, and 0.781 for mobile phase II. The plant extracts MM, BA, CO, JC, and CA had two bands in mobile phase I with clear separation but had single band in mobile phase II. The plant extracts having only one band in either mobile phase suggest a poor solvent system for compounds separation. The TLC profiles provide a characteristic fingerprint of these plants that may also be useful for their identification.

### 3.3. Antioxidant Assays

#### 3.3.1. TPC and TFC

**Figure 1:**
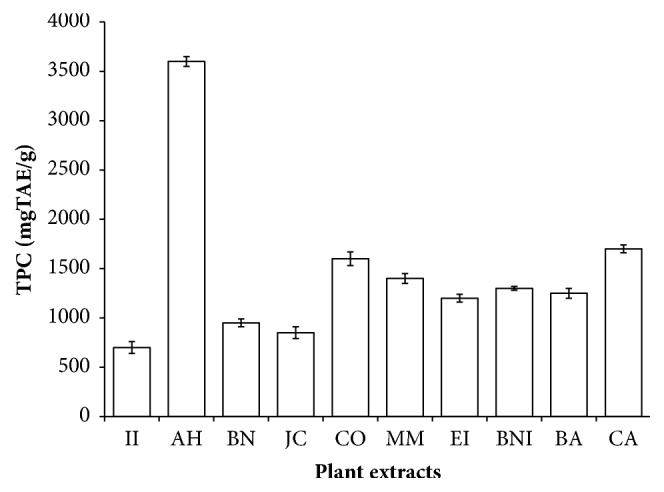
The total phenolic content (TPC) of selected plants used in the management of oral and skin infections in southeast Nigeria. II,* Ipomoea involucrata*; AH,* Acalypha hispida*; BN,* Breynia nivosa*; JC,* Jatropha curcas*; CO,* Chromolaena odorata*; MM,* Macrolobium macrophyllum*; EI,* Eleusine indica*; BNI,* Baphia nitida*; BA,* Burkea africana*; CA,* Cassia alata*; TAE, tannic acid equivalent.

**Figure 2:**
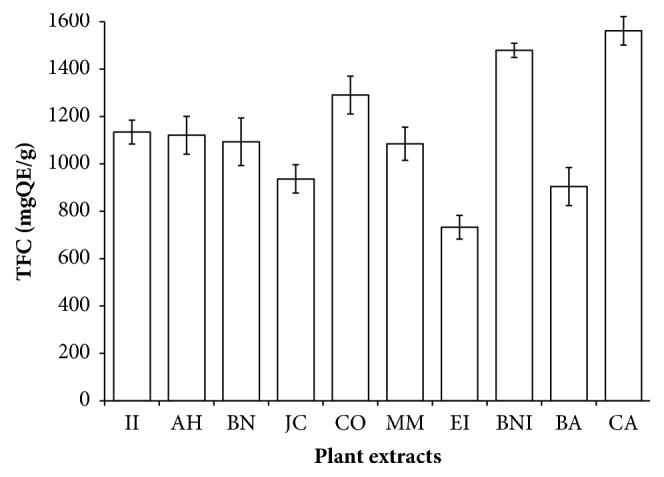
The total flavonoid content (TFC) of selected plants used in the management of oral and skin infections in southeast Nigeria. II,* Ipomoea involucrata*; AH,* Acalypha hispida*; BN,* Breynia nivosa*; JC,* Jatropha curcas*; CO,* Chromolaena odorata*; MM,* Macrolobium macrophyllum*; EI,* Eleusine indica*; BNI,* Baphia nitida*; BA,* Burkea africana*; CA,* Cassia alata*; QE, Quercetin equivalent.

Phenolic compounds are secondary metabolites distributed in fruits, vegetables, nuts, herbs, seeds, and beverages [22]. They are excellent scavengers of free radical that has been implicated in the pathologies of various human diseases, the antioxidant activity is dependent on the structure hydrophobic benzenoid rings and hydrogen-bonding potential of the phenolic hydroxyl groups. In TPC assay, a phenol in plant extracts loses a proton to form phenolate ion, which reduces Folin Ciocalteu reagent, and the resulting changes are measured spectrophotometrically at 765 nm [13]. The content of phenol is reported in mg of tannic acid equivalent (TAE)/mL of extract. As shown in Figure 1, the total phenolic contents of absolute methanol crude extract of these botanicals range from 700 to 3600 mgTAE/g. The AH extract had the highest concentration followed by CA, CO, MM, BNI, BA, EI, BN, and JC while the least concentration was recorded in II extract. Moreover, the Folin Ciocalteu reagent (FCR) has been used to measure other compounds such as ascorbic acid, amino acids, and sugars; hence it may not provide a concise amount of the phenolic compounds present in these plants. Flavonoids are increasingly consumed in a significant amount in daily diets worldwide due to their various health benefits to man [23, 24]. In TFC assay, aluminum ion in the reacting mixture forms complexes with the C- 4 keto and either C- 3 or C- 5 hydroxyl, or with ortho hydroxyl groups in the A or B ring of flavonoids detected in the plant extracts [14]. As shown in Figure 2, the total flavonoid content was recorded in the plant extracts ranging from 750 to 1500 mgQE/g as extrapolated from the standard quercetin curve. The CA extract had the highest concentration followed by BNI, CO, II, AH, BN, MM, JC, and BA while the lowest concentration was recorded in EI extract. TPC is found higher than the TFC in some of the extracts supporting the fact that most flavonoids are also phenolics. According to our results, all the plant extracts are rich source of phenolic compounds. The composition of TPC and TFC registered in these plants may be difficult to compare with others reported elsewhere due to many factors including differences from species to species, maturity stage, plant parts, and assay methods, harvesting time, standard drugs, and geographical location.

#### 3.3.2. Reducing Power (RP)

**Figure 3:**
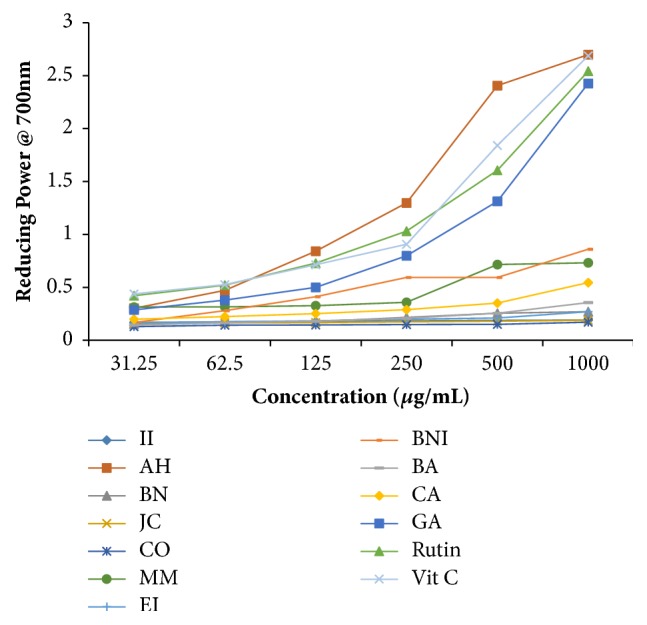
The ferric reducing antioxidative capacity of selected plant extracts used in the management of oral and skin infections in southeast folklore medicine of Nigeria. II,* Ipomoea involucrata*; AH,* Acalypha hispida*; BN,* Breynia nivosa*; JC,* Jatropha curcas*; CO,* Chromolaena odorata*; MM,* Macrolobium macrophyllum*; EI,* Eleusine indica*; BNI,* Baphia nitida*; BA,* Burkea africana*; CA,* Cassia alata*; GA, gallic acid; Vit C, vitamin C (standard antioxidants).

Ferric reducing power is a global method often used to assess the capacity of antioxidants to donate either an electron or hydrogen atom to unpaired electron [25]. In reducing power assay, plant extracts exert antioxidant activities via reduction of Fe^3+/^ferric cyanide complex to Fe^2+^ (ferrous form) resulting in changing the green colour to blue and sometimes Perl's Prussian blue colour depending on the antioxidant potential [10]. As shown in Figure 3, the reducing power of the methanol crude extracts and the standard gallic acid, rutin, and ascorbic acid increased with increase in concentration. Higher absorbance at 700 nm can be read after potassium ferricyanide reaction with ferric chloride. At 1000 *μ*g/mL, the extracts from AH, BNI, MM, CA, BA, EI, and BN had absorbance value of 2.70, 0.86, 0.73, 0.55, 0.36, 0.27, and 0.27, respectively, while that of AH is higher than the standard rutin (2.54) but comparable well with gallic acid (2.75) and ascorbic acid (2.69). EI and BN extracts had a similar trend of ferric reducing power though significantly lower as compared with the standard drugs. AH extract exhibits remarkable antioxidant capacity (RP) correlating with its richness in phenolic and flavonoid contents. It is reasonable to suggest that methanol selectively extracts antioxidant principles from these botanicals based on their polarities and chemical structures which are reflected in plant extracts with high TFC and TPC having minimal RP [26]. We have shown that some plant extracts, in particular, AH, BNI, MM, and CA extracts, may serve as an excellent candidate to minimize skin and oral infections caused by oxidative stress as a result of imbalance of free radicals generation and natural antioxidants in the saliva or wound area. According to our results, the increasing RP of the plant extracts correlates with their TPC and TFC. This study, at least in part, lends credence to the ethno-therapeutic use of some plant as natural antioxidant in the management of skin or oral infections in the study area.

#### 3.3.3. FRSA of Plant Extracts

**Figure 4:**
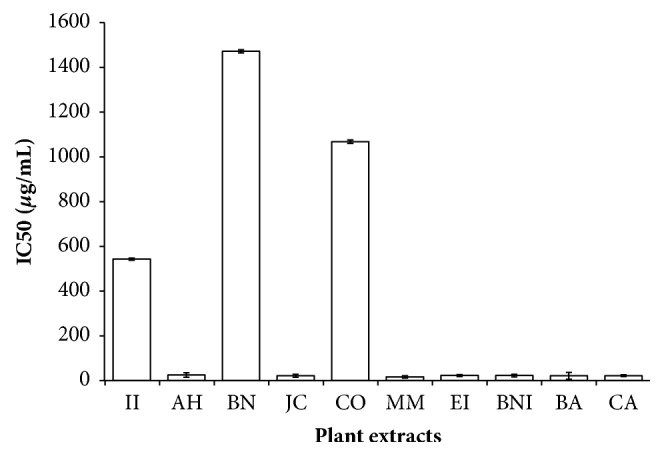
DPPH free radical scavenging activity of selected plant extracts used in the management of oral and skin infections in southeast folklore medicine of Nigeria. II,* Ipomoea involucrata*; AH,* Acalypha hispida*; BN,* Breynia nivosa*; JC,* Jatropha curcas*; CO,* Chromolaena odorata*; MM,* Macrolobium macrophyllum*; EI,* Eleusine indica*; BNI,* Baphia nitida*; BA,* Burkea africana*; CA,* Cassia alata*.

**Figure 5:**
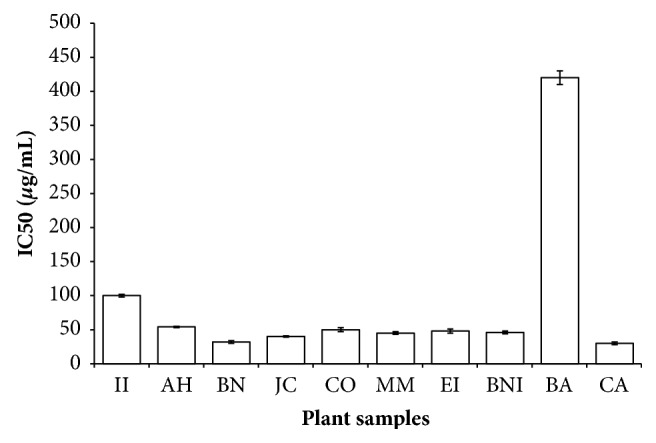
ABTS free radical scavenging activity of selected plant extracts used in the management of oral and skin infections in southeast folklore medicine of Nigeria. II,* Ipomoea involucrata*; AH,* Acalypha hispida*; BN,* Breynia nivosa*; JC,* Jatropha curcas*; CO,* Chromolaena odorata*; MM,* Macrolobium macrophyllum*; EI,* Eleusine indica*; BNI,* Baphia nitida*; BA,* Burkea africana*; CA,* Cassia alata*.

Skin and oral cavity exposed to an array of oxidants such as smokes from cigarettes, chemicals, UV light, nicotine, and alcohol that are directly or indirectly leading to the production of reactive oxygen species (ROS) are known to be responsible for some common human diseases [27]. These free radicals in particular hydroxyl and superoxide anions are highly reactive molecules derived from the metabolism of oxygen. Although they play an important role in the biochemical, immunological, and physiological processes, it could be deleterious to vital organs if excessively accumulated in the human body [28]. Food and pharmaceutical industries discovered that medicinal plants containing phenol and flavonoid compounds are essential in the development of novel drugs against stress-induced diseases [28]. In the present investigation, the concentration (IC_50_) at which 50% of free radical scavenged was calculated and extrapolated from the plot of percentage inhibition versus plant extract concentration. The herbal drugs exhibited robust DPPH radical scavenging activity in a concentration-dependent manner having IC_50_ ranging from 20.25 to 710 *μ*g/mL. As shown in Figure 4, MM, BA, EI, JC, AH, BNI, and CA extracts had a potent DPPH radical scavenging activity corresponding to their TPC, TFC, and RP. The plant extracts scavenged ABTS radical by the decrease in its absorbance at 734 nm. All the plant extracts exhibited strong ABTS radical scavenging capacities in the following order: BN > JC > CA > MM > BNI > EI > CO > AH > II > BA having IC_50_ values ranging from 25 to 400 *μ*g/mL (Figure 5). DPPH and ABTS radical scavenging activities of these herbal remedies displayed a positive correlation with the working concentrations. It is obvious that most of the plant extracts tested in this work possess the ability to quench the free radicals generation via electron or proton donation demonstrated by the termination of radical chain reaction [28]. Although both DPPH and ABTS radicals are electron-transfer based methods, however, it is apparent that ABTS method seems to be more sensitive than DPPH radical assay, possibly due to their solubility and diffusivity as shown in the IC_50_ values [10]. It is worth noting that BA extract with strong DPPH radical scavenging effect had a moderate ABTS radical scavenging capacity which can be explained by the partial ionization of the extract in the test medium [29]. Since ROS promotes tissue damage and epithelial inflammation via exposure to sunlight, radiation, or smoke as a result the antioxidant capacity demonstrated in this work may support the traditional use of these plants against the influence of inflammatory markers and oxidants in the skin and oral cavity [29]. Based on the results of the three antioxidant assays, it is obvious that MM, AH, BNI, EI, and CA extracts had the best FRSA indicating their importance in converting free radicals into more stable products and then terminate the free radical chain reaction. Our study showed that the methanol crude extracts from these plants could protect against free radical damage in the mouth such as human gingival, periodontal tissues, and certain skin infections [5].

#### 3.3.4. Antibacterial Activity of Plant Extracts

Pathogenic bacteria such as* Staphylococcus aureus*,* Escherichia coli*,* Klebsiella pneumoniae*, and* Proteus mirabilis* play a significant role as the primary cause of skin and oral infections [30].* Staphylococcus aureus* is a part of normal skin known to be the most problematic in the cause of skin infections as a result of their resistance mechanisms to first-line antibiotics [30]. Although the presence of this organism as a resident oral floral is controversial, nonetheless, MSSA and MRSA have been isolated from oral rinse and tongue swab in patients with oral infections [31].* Escherichia coli* are Gram-negative bacteria known to be notorious for their resistant mechanisms towards the existing arsenal of antibiotics. Oral infections such as edematous and erythematous and skin infections like omphalitis, surgical site infections, burn, necrotizing fasciitis, and cellulitis at all age groups are associated with enterobacterium* E. coli* [32].* Klebsiella pneumoniae* is a Gram-negative, encapsulated, facultative anaerobic, and rod-shaped bacterium found in the normal flora of the mouth, skin, and intestines. This bacterium is responsible for the prolonged surgical wound, biliary tract wound, and urinary tract infections and it could pose a serious health risk when resistant strains invade the throat, mouth, or intestines [33]. Villegas et al. [34] detected a CTX-M-12 *β*-lactamase producing* K. pneumoniae* new plasmid-mediated ESBLs highly resistant to ceftazidime from a patient with hospital-acquired wound infection.* Proteus mirabilis* is one of the most common enterobacteria after* E. coli* identified as Gram-negative, motile, and rod-shaped bacterium. It is found in clinical specimen implicated in the skin, respiratory tract, and bacteraemia as well as burns, wounds, and urinary tract infections [35]. All these pathogenic multidrug-resistance bacteria are causative agent in a periodontal, lips ulcer, white sponge nevus, and skin infections [36].

#### 3.3.5. Susceptibility Test and MIC Determination

The result of disc diffusion assay showed the diameter of inhibition of bacteria growth classified as ≤ 15 (resistant) and ≥ 16 (susceptible) according to CLSI guideline [37]. As shown in Table 4,* K. pneumoniae* and* E. coli* are more susceptible to the treatment of* Acalypha hispida* (AH) having a zone of inhibition (ZI) 21 and 17 mm, respectively. BN and EI extracts inhibit the growth of* P. mirabilis* and* E. coli* having the same zone of inhibition 17 mm corresponding to the report of Al-Zubairi et al. [38]. The data obtained for the methanol crude extracts from AH, BN, and EI show their selective inhibition of the test bacteria growth. The plant extracts with ZI above 13 mm against our panel of bacterial strains were selected for MIC determination. Here the methanol crude extracts exhibit MIC values of 256 to > 512 *μ*g/mL against the test bacterial strains but were less active as compared to the standard ciprofloxacin 0.03 - 0.06 *μ*g/mL (Table 5). Ciprofloxacin is an active broad-spectrum antibiotic use in the treatment of urinary tract infections, prostatitis, and acute cystitis via interfering with bacterial DNA replication and transcription through inhibition of DNA gyrase or topoisomerase [39]. All the plant extracts demonstrated a mild to weak antibacterial activity based on the classification of Rios and Recio [40] who considered antibacterial activity of the crude plant extract significant (MIC < 100 *μ*g/mL), moderate (100 < MIC = 512 *μ*g/mL), or weak (MIC > 512 *μ*g/mL). The herbal remedies showed moderate inhibition of bacterial growth except for* K. pneumoniae* and* P. mirabilis* towards BN and AH extracts, respectively. BN and EI extracts were more active against* S. aureus* and* P. mirabilis*; CA and CO extracts were more active against all the test bacterial strains except* S. aureus*. Previous work reported by Amadi et al. [41] confirmed the weak antibacterial activity exhibited by BN extract against* K. pneumoniae*. The antibacterial activity demonstrated by AH extract against our panel of bacterial strains contradicts that of Aboaba and Yeye [42] who reported MIC value of 100 mg/mL against* E. coli*,* S. aureus*, and* K. pneumoniae*. It is interesting to note that* K. pneumoniae*,* E. coli*, and* P. mirabilis* that confer resistance to the extended spectrum cephalosporin and penicillin [43] are more susceptible to the treatment with CA and CO extracts. Furthermore, the observed antibacterial activity exhibited by EI, CA, and CO extracts against the superbugs of modern day healthcare [38] is worth noting which may perhaps attribute to a wide variety of secondary metabolites especially phenolics and flavonoids that have been reported with potent antimicrobial properties [44]. Among the three extracts,* Eleusine indica* has IC_50_ > 30 *μ*g/ mL towards MCF-7, HT-29, and CEM-SS human cancer cell lines suggesting that continued exposure to this herbal remedy may alter human organs [38]. The probable mechanism of action of these plants is not known but it could be attributed to the leakage of the cell wall or alteration in the membrane permeability or inhibition of enzyme gyrase, DNA synthesis, and protein synthesis [45]. Further researches are needed to identify the active ingredients, curare effect, and the molecular mechanisms of these plants.

## 4. Conclusion

The results of this study, therefore, at least in part provide scientific evidence for the traditional use of these medicinal plants in ethnomedicine by the dwellers of Ebem-Ohafia communities in the treatment of skin and oral infections. The free radicals scavenging activity, moderate to high TPC, TFC, and the mild antibacterial properties demonstrated by certain plant extracts are worth harnessing for proper evaluation and integration into the primary healthcare system. Further fractionation to identify the single compound responsible for the observed activities is highly recommended. Encouragingly, BN, CA, CO, and AH extracts with potent antioxidant and antibacterial properties did not exhibit any significant toxic effect as reported by other researchers and thus substantiate its safe use as complementary and alternative therapy for the treatment or prevention of human diseases.

## Figures and Tables

**Table 1:** Medicinal plants used in the management of skin or oral infections in southeastern part of Nigeria.

Plant Name	Family	Local name	Part used	Infection	Traditional uses	Method of preparation
*Macrolobium macrophyllum*	Fabaceae	Anthonotha macrophylla	Leaf	Skin	Treating of wound and cut	Squeeze the leave and place on the affected area of the skin
*Jatropha curcas*	Euphorbiaceae	Barbados nut	Leaf	Skin/oral	Treatment of boils	Boil the leave with water. Place the hot leave on the affected area of the skin
* Eleusine indica*	Poaceae	Yard grass	Root	Skin	Treatment of Ringworm	Use the root and scrub on affected area of the skin
*Cassia alata*	Caesalpiniaceae	Ringworm bush	Leaf	Skin	Treatment of Ringworm	Scrub the leave on affected area of the skin
*Chromolaena odorata*	Asteraceae	Siam weed	Leaf	Skin	To stop bleeding and rashes	Squeeze the leave and rub on affected area
*Psidium guajava *(L)	Myrtaceae	Guava	Leaf	Skin	Treatment of skin burns, scrapes, sunburn, wounds, psoriasis, eczema, and others	Crushed guava leaf or boiled leaves are applied directly to the affected skin, providing relief, and are believed to improve healing
*Bryophyllum pinnatum (Lam.) Oken.*	Crassulaceae	Miracle leaf, Dog's liver	Leaf	Skin/ oral	Treatment of boil, wound, sore or cut,	Crush leaf and apply juice on the wound surface
*Ficus asperifolia *Miq.	Moraceae	False thistle	Leaf latex	Skin	Treatment of skin rashes and itches	Applied as poultice to the skin itches
*Acanthus montany (T. Anderson)*	Acanthaceae	Bear's breeches	Stem bark	Skin	Treatment of wounds and skin diseases	Wood bark is crushed and soaked it alcohol, and extract applied to wounds and skin
*Allium sativum L.*	Liliaceae	Garlic	Bulb juice	Skin/ oral	Used to condition the skin, chewed for cough and sore throat	The juice is mixed with lotions for skin conditioning. Fresh bulb is chewed or boiled in water and drink decoction for cough
*Emilia sonchifolia *(L.)	Asteraceae	Scarlet tassel	Leaf	Skin	Treatment of Skin troubles, abscess and bruises	Crushed leaves were pressed and used to cover the wound or bruised surfaces
*Sida acuta* Burm.f	Malvaceae	Broom weed	Leaf	Skin	Treatment of jaundice, sore, ulcer	Poultices made from boiled leaves are applied on the affected parts
*Ricinus communis* Linn.	Euphorbiaceae	Castor bean	Leaf, root and seed	Skin	Treatment of skin infection and detoxification	Apply castor oil on the affected area to relieve skin infection
*Citrus limon *(L.) Burm.f. (pro.sp.)	Rutaceae	Limon	Leaf, fruit and seed	Skin	Treatment of flu, headache, cold and oral infections	Mix equal quantities of Limon juice and holy basil, keep it under the sun till becomes thick; apply this on face
*Allium cepa .L*	Liliaceae	Onions	Leaf and bulb	Skin	Treatment of periodontitis and cold	The bulb is macerated and mixed with water and then taken orally
*Breynia nivosa*	Euphorbiaceae	Ice plant	Leaf	Oral	Treating of toothache	Boil the leave with water, then take the water.
*Ipomoea involucrata*	Convolvulaceae	Water convolvulus	Leaf and stem	Oral	Treatment of oral infection	Mix the leave with ugbaga tree and boil with water and drink the water
*Burkea africana*	Caesalpinioideae	Icheku	Leaf	Oral	Treatment of oral disease	Boil the leave with water and take, but don't swallow it.
*Baphia nitida*	Papilionaceae	Cam wood	Leaf or stem	Oral	Strengthens the teeth	Chew the stem or scrub the leave on the teeth
*Gongronema latifolium*	Asclepiadaceae	Utazi	Leaf	Oral	Treatment of cough and diabetes	Crush the leaf with mortar and mix with ginger or garlic and drink
*Anona senegalensis*	Annonaceae	Uburu ocha	Root	Oral	Treatment of malaria and mouth infection	Boil the roots and drink
*Abrus precatorius*	Papilionoideae	Crab's eyes	Seed and root	Oral /skin	Treatment of cough, skin care, wound	Grind the root and apply the poultice on the affected skin area. Also crushed seeds can be taken orally
*Acanthus montanus*	Acanthaceae	False thistle	Leaf and root	Oral and Skin	Treatment of furuncles, lesions and urinary infections	Boil the root and apply the poultice to the affected part of the body. For infections, boil the root and leaves and drink.
*Piper umbellatum*	Piperaceae	Njamuja	Leaf	Oral	Treatment of peptic ulcers	Crush the leaves and soak in warm or hot water and drink
*Euphorbia hirta*	Euphorbiaceae	Asthma weed	Whole plant	Oral	Treatment of wounds	Leaves are macerated and applied to affected part
*Ficus burkei (Miq.) Miq.*	Moraceae	Sand paper	Leaf	Oral	Treatment of hypertension	Leaves are boiled and taken orally
*Gmelina arborea*	Verbenaceae	Gmelina	Stem and leaf	Oral	Treatment of gonorrhea, piles, abdominal pains, burning sensations, fever, mouth wound and diabetes. Snake-bite and scorpion sting.	Leaves ground into paste with water are applied to the forehead for headache in fevers. Boiled stems are taken orally
*Lophira alata*	Ochnaceae	Ekki	Stem and leaf	Oral	Treatment of fever, mouth wound, and malaria	Boil the leaves and stem and take orally
*Nauclea latifolia*	Rubiaceae	Uburu-inu	Stem and leaf	Oral	It is used in the treatment of malaria, epilepsy, mouth wound, anxiety, pain, fever.	Boil the roots and drink
*Annona muricata*	Annonaceae	Sour sop	Leaf	Oral	Treatment of cancer and mouth wound infections.	Crush or boil the leaves and take orally
*Psidium guajava*	Myrtaceae	Guava	Leaf	Oral	Treatment of periodontal disease, burns, scrapes, sunburn, wounds, psoriasis, eczema, and others	Leaves are crushed and taken orally
*Astragalus membranaceus*	Fabaceae	Bei qi	Stem and leaf	Oral	Treatment of cold, upper respiratory infections, allergies, fibromyalgia, anemia, HIV/AIDS.	Leaves are crushed and taken orally and can also be applied to skin
*Buchholzia coriacea*	Capparaceae	Wonderful kola	Leaf	Oral	It is used traditionally for treating diabetes, hypertension, rheumatism, cold, cough, and catarrh	Boil leaves and take orally
*Gnetum africanum*	Gnetacea	Afang	Leaf	Oral	Enlarged spleen, sore-throat and cathartic	Leaves can be boiled or cooked and taken orally
*Anoridium manni*	Annonaceae	Ewuro -igbo	Stem bark	Oral	Treatment of Diarrhea, cough, fever, rheumatism	The bark is crushed and boiled and then taken orally
*Funtumia africana*	Apocynaceae	Akorie, mbamiri	Leaf, stem, root	Skin	treatment of constipation, wounds, weak bladder, jaundice	Whole plant is boiled and taken orally. Also the crushed leaves are applied to the affected skin area
*Commiphora africana*	Burseraceae	Turari, dashi	Roots, fruits	Oral	Treatment of whooping cough, bronchitis	Roots are macerated and taken orally
*Dacryodes edulis*	Burseraceae	Elemi, ube	Bark, roots, fruits	Skin	Treatment of Jiggers, Skin diseases, Elephantiasis	The leaves are eaten raw with kola nut as an antiemetic. Leaf-sap is instilled into the ear for ear-trouble, and a leaf-decoction is prepared as a vapour-bath for feverish stiffness with headache
*Combretum mucronatum*	Combretaceae	Farar geza	Roots and leaf	Skin	Treatment of skin infection	The root and leaf are macerated and applied to affected skin area
*Cleome ciliata*	Capparaceae	Ekuya	Leaf, seeds	Skin	Treatment of Convulsion, wounds, sores	Seeds are crushed and the poultice applied to the affected area. The macerated leaves after boiled are taken orally
*Ageratum conyzoides*	Asteraceae	Urata	Whole plant	Skin	Treatment of wound, Ulcer, Sleeping sickness, Eyewash	The plant is macerated and applied to affected skin area.
*Aspilia africana*	Asteraceae	Yunyun, Kalankuwa	Leaf, flower	Skin	Treatment of skin rashes, cleaning sores, corneal opacities	The flower is squeezed and applied to affected skin area.
*Centaurea perrottetii*	Asteraceae	Danyi	Whole plant	Skin	Treatment of skin infections, Syphilis	The flower is squeezed and a poultice applied to affected skin area.
*Chrysanthellum indicum*	Asteraceae	Abilere, oyigi	Whole plant	Skin	Treatment of boils, gonorrhea, Jaundice	The plant is crushed and the poultice applied directly to the skin area
*Elephantopus scaber*	Asteraceae	Elephant foot	Leaf, root	Oral	Treatment of fever, cough	Leaf and root are boiled together and taken orally
*Emilia coccinea*	Asteraceae	Odundun	Leaf, sap and root	Skin	Treatment of ulcer, hernia, measles	Whole plant is squeezed and sap taken orally
*Acalypha godseffiana*	Euphorbiaceae	Jinwinini	Leaf	Skin	Treatment of skin infections, antimicrobials	Leaf is macerated and taken orally as well as applied to affected skin area
*Bridelia ferruginea*	Euphorbiaceae	Iri, kirni	Leaf, stem, bark root	Oral	Treatment of Insomnia, mouth wash, gonorrhea	Whole plant is boiled and taken orally
*Euphorbia heterophylla*	Euphorbiaceae	Egele	Leaf, roots	Skin	Treatment of Skin diseases	Leaf and root are macerated and the poultice applied to the affected skin area
*Euphorbia hirta*	Euphorbiaceae	Nonon kurciya, odne	Whole plant exudate	Oral	Treatment of Asthma, cough, shape of breasts	The whole plant is macerated and taken orally
*Euphorbia lateriflora*	Euphorbiaceae	Oro, were	Leaf exudate	Skin	Treatment of dermatitis, constipation	The leaf is squeezed and the poultice applied to the affected skin area
*Cajanus cajan*	Fabaceae	Orela	Leaf, seed	Skin and oral	Treatment of Smallpox and mouth wash	Leaf is squeezed and applied to the affected area.
*Daniellia thurifera*	Fabaceae	Iya	Stew-wood dust	Skin	Treatment of scabies	The bark is macerated and applied to the affected skin area
*Dialium guineense*	Fabaceae	Tasmiyar kumi	Leaf, bark, fruit and twig	Oral	Treatment of Bronchitis, cough and diuretic	The leaf and bark are crushed and boiled together. The mixture is then taken orally.
*Acacia nilotica*	Fabaceae	Baani, gabaruwa	Fruit, bark, exudate	Skin	Treatment of skin diseases, fungal infections	The exudates are squeezed from the bark and applied directly to the affected skin area
*Napoleona imperialis*	Lecythidaceae	Mabungi	Twigs	Oral	Treatment of Asthma, cough	Twigs are macerated and taken orally
*Allium sativum*	Liliaceae	Ayo, ayuu	Bulb	Oral	Treatment of fever, cough, asthma, antimicrobial	The bulb is macerated and taken orally
*Hibiscus asper*	Malvaceae	Isapa, dangiraa	Leaf	Oral and skin	Treatment of cough, wounds and diuretic	Leaf is boiled and taken orally
*Hibiscus rosasinensis*	Malvaceae	Kekeke, ireagu	Leaf, stem, flower	Skin	Treatment of influenza, wounds, and diuretic	Whole plant is macerated and taken orally. The leaf is squeezed and applied in affected skin area
*Hibiscus sabdariffa*	Malvaceae	Sobo, gurguzu	Leaf, flower	Oral	Treatment of Diuretics, cough and dressing wounds	Leaf is boiled and taken orally.
*Ficus elegans*	Moraceae	Asoro	Leaf	Skin/oral	Treatment of Pile, constipation and craw-craw	Leaf is squeezed and applied to the affected area

**Table 2:** The list of family, number of the plant species, and their percentage occurrence.

Family	Number of plant species	% Occurrence
Fabaceae	7	11.47
Euphorbiaceae	11	18.03
Asteraceae	7	11.47
Myrtaceae	4	6.56
Moraceae	3	4.92
Malvaceae	4	6.56
Liliaceae	2	3.28
Capparaceae	2	3.28
Annonaceae	3	4.92
Combretaceae	1	1.64
Burseraceae	2	3.28
Apocynaceae	1	1.64
Gnetaceae	1	1.64
Lecythidaceae	1	1.64
Verbenaceae	1	1.64
Ochnaceae	1	1.64
Rubiaceae	1	1.64
Piperaceae	1	1.64
Acanthaceae	2	3.28
Papilionoideae	2	3.28
Asclepiadaceae	1	1.64
Caesalpiniaceae	2	3.28
Convolvulaceae	1	1.64
Rutaceae	1	1.64
Crassulaceae	1	1.64
Poaceae	1	1.64

**Table 3:** Thin layer chromatography retention factor using mobile phase CHL: ET (mobile phase I) and Tol  : EA  : AA (mobile phase II).

S/N	Plant Extract	CHL: ET (6:4)	Tol: EA: AA (5:4:1)
1	*Baphia nitida*	1	0.74
2	*Macrolobium macrophyllum*	0.706, 0.941	0.695
3	*Chromolaena odorata*	0.471, 0.647	0.667
4	*Jatropha curcas*	0.353, 0.941	0.648
5	*Cassia alata*	0.118, 0.882	0.686
6	*Ipomoea involucrata*	0.956	-
7	*Acalypha hispida*	0.667, 0.833, 0.989	0.381, 0.648, 0.781
8	*Breynia nivosa*	0.989	0.762
9	*Burkea africana*	0.644, 1	0.752
10	*Eleusine indica*	0.978	0.743

CHL, chloroform; ET, ethanol; Tol, toluene; EA, ethylacetate; AA, acetic acid

**Table 4:** Zone of bacterial inhibition by the selected plant extracts use in the management of oral and skin infections.

Diameter of inhibition (mm)					

S/N	Plant samples	*K. pneumoniae*	*P. mirabilis*	*S. aureus*	*E. coli*

1	*Cassia alata*	15 ± 1.41	9 ± 0.42	15 ± 0.83	13 ± 0.80
2	*Breynia nivosa*	8 ± 0.52	17 ± 1.00	8 ± 0.08	15 ± 1.02
3	*Burkea africana*	NI	11 ± 0.84	13 ± 0.72	NI
4	*Baphia nitida*	8 ± 0.50	13 ± 1.22	8 ± 0.51	NI
5	*Ipomoea involucrata*	8 ± 0.52	NI	8 ± 0.50	13 ± 0.52
6	*Jatropha curcas*	NI	9 ± 0.30	9 ± 0.62	13 ± 0.72
7	*Acalypha hispida*	21 ± 1.63	NI	11 ± 0.09	17 ± 1.32
8	*Chromolaena odorata*	8 ± 0.42	8 ± 0.35	9 ± 0.09	15 ±1.20
9	*Eleusine indica*	8 ± 0.42	8 ± 0.35	NI	17 ± 1.00
10	*Macrolobium macrophyllum*	NI	NI	8 ± 0.04	15 ± 1.23
11	Ciprofloxacin	-	-	≤16(R); ≥17(S)	≤16(R);≥20(S)

NI indicates no zone of inhibition; S, sensitive; R, resistance

**Table 5:** Minimum inhibitory concentration (MIC) of selected medicinal plants against bacterial strains associated with oral and skin infections.

Plants	SA	KP	PM	EC
	MIC (*µ*g/mL)			
*Acalypha hispida*	512	256	>512	512
*Cassia alata*	512	256	256	256
*Breynia nivosa*	256	>512	256	512
*Eleusine indica*	256	512	256	512
*Chromolaena odorata*	512	256	256	256
Ciprofloxacin	0.03	0.03	0.03	0.03

SA, *S. aureus*; KP, *K. pneumoniae*; PM, *P. mirabilis*; EC, *E. coli*.

## Data Availability

The data used to support the findings of this study are available from the corresponding author upon request.

## References

[1] World Health Organization (2012). Oral health. *Fact sheets*.

[2] Dewhirst F. E., Chen T., Izard J. (2010). The human oral microbiome. *Journal of Bacteriology*.

[3] Falsetta M. L., Klein M. I., Colonne P. M. (2014). Symbiotic relationship between *Streptococcus mutans* and *Candida albicans* synergizes virulence of plaque biofilms *in vivo*. *Infection and Immunity*.

[4] Shinde A., Ganu J., Naik P. (2012). Effect of Free Radicals & Antioxidants on Oxidative Stress: A Review. *Journal of Dental and Allied Sciences*.

[5] Hershkovich O., Shafat I., Nagler R. M. (2007). Age-related changes in salivary antioxidant profile: possible implications for oral cancer. *Journals of Gerontology, Series A: Biological Sciences and Medical Sciences*.

[6] Jagtap S., Yavankar S., Pardesi K., Chopade B. (2010). Production of bioemulsifier by Acinetobactersp. from healthy human skin of tribal population. *Indian Journal of Experimental Biology*.

[7] Limoli D. H., Rockel A. B., Host K. M. (2014). Cationic Antimicrobial Peptides Promote Microbial Mutagenesis and Pathoadaptation in Chronic Infections. *PLoS Pathogens*.

[8] Al-Ahmad A., Ameen H., Pelz K. (2014). Antibiotic resistance and capacity for biofilm formation of different bacteria isolated from endodontic infections associated with root-filled teeth. *Journal of Endodontics*.

[9] Badria F. A., Zidan O. A. (2004). Natural products for dental caries prevention. *Journal of Medicinal Food*.

[10] Oyedemi S. O., Oyedemi B. O., Ijeh I. I., Ohanyerem P. E., Coopoosamy R. M., Aiyegoro O. A. (2017). Alpha-amylase inhibition and antioxidative capacity of some antidiabetic plants used by the traditional healers in Southeastern Nigeria. *The Scientific World Journal*.

[11] Vogt T. (2010). Phenylpropanoid biosynthesis. *Molecular Plant*.

[12] Ghaffari H., Ghassam B. J., Nayaka S. C., Ramachandra Kini K., Prakash H. S. (2014). Antioxidant and neuroprotective activities of *Hyptis suaveolens* (L.) Poit. against oxidative stress-induced neurotoxicity. *Cellular and Molecular Neurobiology*.

[13] Chang C., Yang M., Wen H., Chern J. (2002). Estimation of total flavonoid content in propolis by two complementary colometric methods. *Journal of Food and Drug Analysis*.

[14] Yen G. C., Chen H. Y. (1995). Antioxidant activity of various tea extracts in relation to their antimutagenicity. *Journal of Agricultural and Food Chemistry*.

[15] Tariq A., Athar M., Ara J., Sultana V., Ehteshamul-Haque S., Ahmad M. (2015). Biochemical evaluation of antioxidant activity in extracts and polysaccharide fractions of seaweeds. *Global Journal of Environmental Science Management*.

[16] Re R., Pellegrini N., Proteggente A., Pannala A., Yang M., Rice-Evans C. (1999). Antioxidant activity applying an improved ABTS radical cation decolorization assay. *Free Radical Biology & Medicine*.

[17] National Committee for Clinical Laboratory Standards (2002). Performance Standards for antimicrobial susceptibility testing. *8th Informational Supplement*.

[18] Smith E. E., Williamson E., Zloh M., Gibbons S. (2005). Isopimaric acid from *Pinus nigra* shows activity against multidrug-resistant and EMRSA strains of *Staphylococcus aureus*. *Phytotherapy Research*.

[19] Farnsworth N. R. (2007). Ethnopharmacology and drug development. *Ciba Foundation Symposium*.

[20] Judd W. S., Campbell C. S., Kellogg E. A., Stevens P. F., Donoghue M. J. (2002). *Plant Systematics: A Phylogenetic Approach*.

[21] Wink M. (2010). *Functions of Plant Secondary Metabolites and their Exploitation in Biotechnology*.

[22] Cheynier V. (2005). Polyphenols in foods are more complex than often thought. *The American Journal of Clinical Nutrition*.

[23] Hollman P. C. H., Katan M. B. (1999). Dietary flavonoids: Intake, health effects and bioavailability. *Food and Chemical Toxicology*.

[24] Hubbard G. P., Wolffram S., Lovegrove J. A., Gibbins J. M. (2004). Ingestion of quercetin inhibits platelet aggregation and essential components of the collagen-stimulated platelet activation pathway in humans. *Journal of Thrombosis and Haemostasis*.

[25] Kou M.-C., Chiou S.-Y., Weng C.-Y., Wang L., Ho C.-T., Wu M.-J. (2013). Curcuminoids distinctly exhibit antioxidant activities and regulate expression of scavenger receptors and heme oxygenase-1. *Molecular Nutrition & Food Research*.

[26] Juan M.-Y., Chou C.-C. (2010). Enhancement of antioxidant activity, total phenolic and flavonoid content of black soybeans by solid state fermentation with *Bacillus subtilis* BCRC 14715. *Food Microbiology*.

[27] Oyedemi S. O., Afolayan A. J. (2011). In vitro and in vivo antioxidant activity of aqueous leaves extract of *Leonotis leonurus* (L.) R. Br. *International Journal of Pharmacology*.

[28] Temraz A., El-Tantawy W. H. (2008). Characterization of antioxidant activity of extract from Artemisia vulgaris. *Pakistan Journal of Pharmaceutical Sciences*.

[29] Fridovich I. (1999). Fundamental aspects of reactive oxygen species, or what's the matter with oxygen?. *Annals of the New York Academy of Sciences*.

[30] Wilson D. J., Gonzalez R. N., Das H. H. (1997). Bovine mastitis pathogens in New York and Pennsylvania: prevalence and effects on somatic cell count and milk production. *Journal of Dairy Science*.

[31] Smith A. J., Jackson M. S., Bagg J. (2001). The ecology of staphylococci in the oral cavity: a review. *Journal of Medical Microbiology*.

[32] Moet G. J., Jones R. N., Biedenbach D. J., Stilwell M. G., Fritsche T. R. (2007). Contemporary causes of skin and soft tissue infections in North America, Latin America, and Europe: report from the SENTRY antimicrobial surveillance program (1998–2004). *Diagnostic Microbiological Infectious Diseases*.

[33] Ryan K. J., Ray C. G. (2004). *Sharris Medicinal Microbiology*.

[34] Villegas M. V., Correa A., Perez F. (2004). CTX-M-12 *β*-lactamase in a *Klebsiella pneumoniae* clinical isolate in Colombia. *Antimicrobial Agents and Chemotherapy*.

[35] Tibbetts R., Frye J. G., Marschall J., Warren D., Dunne W. (2008). Detection of KPC-2 in a clinical isolate of Proteus mirabilis and first reported description of carbapenemase resistance caused by a KPC *β*-lactamase in P. mirabilis. *Journal of Clinical Microbiology*.

[36] Jacobsen S. M., Stickler D. J., Mobley H. L. T., Shirtliff M. E. (2008). Complicated catheter-associated urinary tract infections due to *Escherichia coli* and *Proteus mirabilis*. *Clinical Microbiology Reviews*.

[37] Clinical and Laboratory Standards Institute (2006). Methods for dilution antimicrobial susceptibility tests for bacteria that grow aerobically; approved standard. *CLSI document*.

[38] Al-Zubairi A. S., Abdul A. B., Abdelwahab S. I., Peng C. Y., Mohan S., Elhassan M. M. (2011). *Eleucine indica* possesses antioxidant, antibacterial and cytotoxic properties. *Evidence-Based Complementary and Alternative Medicine*.

[39] Al-Soud Y. A., Al-Masoudi N. A. (2003). A new class of dihaloquinolones bearing N′- aldehydoglycosylhydrazides, mercapto-1,2,4-triazole, oxadiazoline and *α*-amino ester precursors: synthesis and antimicrobial activity. *Journal of the Brazilian Chemical Society*.

[40] Rios J. L., Recio M. C. (2005). Medicinal plants and antimicrobial activity. *Journal of Ethnopharmacology*.

[41] Amadi E. S., Oyeka C. A., Onyeagba R. A., Ugbogu O. C., Okoli I. (2007). Antimicrobial screening of *Breynia nivosus* and *Ageratum conyzoides* against dental caries organisms. *Journal of Biological Sciences*.

[42] Aboaba S. A., Yeye E. (2014). Studies on phytochemical screening, antimicrobial and toxicity effect of the shoot system of *Acalypha segetalis* Müell. Arg. *African Journal of Pure and Applied Chemistry*.

[43] Wahab A., Begum S., Ayub A. (2014). Luteolin and kaempferol from *Cassia alata*, antimicrobial and antioxidant activity of its methanolic extracts. *FUUAST Journal of Biology*.

[44] Lewis K., Ausubel F. M. (2006). Prospects for plant-derived antibacterials. *Nature Biotechnology*.

[45] Kapoor G., Saigal S., Elongavan A. (2017). Action and resistance mechanisms of antibiotics: A guide for clinicians. *Journal of Anaesthesiology Clinical Pharmacology*.

